# ITGβ6 Facilitates Skeletal Muscle Development by Maintaining the Properties and Cytoskeleton Stability of Satellite Cells

**DOI:** 10.3390/life12070926

**Published:** 2022-06-21

**Authors:** Hong Zhang, Yuan Liu, Cencen Li, Weiya Zhang

**Affiliations:** 1 Agricultural Technology Innovation Center in Mountainous Areas of Hebei Province, Hebei Agricultural University, Baoding 071000, China; zhanghong@hbnydx161.wecom.work (H.Z.); liuyuan@hbnydx162.wecom.work (Y.L.); 2 College of Life Sciences, Xinyang Normal University, Xinyang 464000, China; licencen@xynu.edu.cn; 3 National Engineering Research Center for Agriculture in Northern Mountainous Areas, Hebei Agricultural University, Baoding 071000, China

**Keywords:** skeletal muscle, satellite cell, ITGβ6, cell differentiation, cell adhesion, cytoskeleton system

## Abstract

Integrin proteins are important receptors connecting the intracellular skeleton of satellite cells and the extracellular matrix (ECM), playing an important role in the process of skeletal muscle development. In this research, the function of ITGβ6 in regulating the differentiation of satellite cells was studied. Transcriptome and proteome analysis indicated that *Itgβ6* is a key node connecting ECM-related proteins to the cytoskeleton, and it is necessary for the integrity of the membrane structure and stability of the cytoskeletal system, which are essential for satellite cell adhesion. Functional analysis revealed that the ITGβ6 protein could affect the myogenic differentiation potential of satellite cells by regulating the expression of PAX7 protein, thus regulating the formation of myotubes. Moreover, ITGβ6 is involved in muscle development by regulating cell-adhesion-related proteins, such as β-laminin, and cytoskeletal proteins such as PXN, DMD, and VCL. In conclusion, the effect of ITGβ6 on satellite cell differentiation mainly occurs before the initiation of differentiation, and it regulates terminal differentiation by affecting satellite cell characteristics, cell adhesion, and the stability of the cytoskeleton system.

## 1. Introduction

Satellite cells are a type of pluripotent stem cells in skeletal muscle that have the ability of myogenic differentiation. When activated, satellite cells can swiftly enter the cell cycle, trigger the process of proliferation and differentiation, and then participate in the regulation of skeletal muscle development [1,2]. Satellite cells are located in the niche between the myofiber membrane and basal lamina [3]. Extracellular matrix (ECM) is the main component of the satellite cell niche and plays an important role in maintaining satellite cell characteristics and cellular environmental homeostasis [4]. ECM provides a stable microenvironment for the migration, adhesion, proliferation, and differentiation of satellite cells during skeletal muscle development. The balance between intracellular homeostasis in satellite cells and stability of the ECM composition is the basis of normal development of skeletal muscle [5]. Studies have demonstrated that aberrant expression of ECM-related genes could lead to changes in the microenvironment of satellite cells, thus weakening the differentiation ability of satellite cells and ultimately affecting skeletal muscle development [6,7].

The membrane of a satellite cell is in close contact with the basal membrane and myofiber membrane, so the integrity of the membrane structure is crucial for signal transmission inside and outside the cell. Membrane receptors play a key role in intracellular and extracellular signal transmission; one of the most important of them is the integrin family. Integrin proteins, located in cell membranes, not only act as a bridge of signal transduction between intracellular and extracellular parts, but also participate in the regulation of multiple physiological activities in cells by interacting with intracellular signal factors or pathways [8,9,10]. During skeletal muscle development, ECM regulates the activation, proliferation, and differentiation of satellite cells through integrin protein, and satellite cells or muscle cells are connected with the ECM via integrin protein to maintain the stability of the cell structure and microenvironment [4,11]. Studies have shown that integrin proteins could directly regulate the proliferation and differentiation of satellite cells, the remodeling of ECM, and the maintenance of the physiological activities and homeostasis of satellite cells [12,13]. In addition, satellite cells can secrete ECM proteins such as collagen and laminin to form a local microenvironment and maintain their own physiological activities [14,15,16]. Therefore, ECM remodeling regulated by satellite cells is required for the regulation of cell differentiation and skeletal muscle development.

Integrin proteins function as heterodimers consisting of α and β subunits, such as Itgα5β1 [17], α7β1 [12], and α9β1 [18]. Although many studies have reported that different subtypes of integrin proteins are involved in coordinating the differentiation and ECM composition of satellite cells, different subtypes have different ligands and different mechanisms of action in skeletal muscle development. This study will explore the molecular regulatory network of satellite cell differentiation and ECM remodeling and analyze the important role of Itgβ6 in regulating skeletal muscle development.

## 2. Materials and Methods

**Mice.** All the SPF (specific pathogen free)-grade 4-week-old wild-type female C57BL/6 mice used to isolate skeletal muscle satellite cells in this study were obtained from Ex&Invivo Biological Technology Co., Ltd. (Shijiazhuang, China). All animal experiments took place at the biology laboratory and were approved by the Experimental Animal Ethics Committee of Hebei Agricultural University (Approval number: 2021088). The procedures using mice in this project complied with the National Research Council’s Guide for the Care and Use of Laboratory Animals (Institute of Laboratory Animal Resources, Commission on Life Sciences, National Research Council, 1996).

**Isolation and culture of muscle satellite cells.** Satellite cells were isolated from hind-limb muscle tissues obtained from wild-type female C57BL/6 mice aged 4 weeks. Each cell isolation experiment required 10 mice. For the satellite cell isolation method, we referred to that described in our previous study [19]. The specific methods were as follows: The muscle tissues were digested for 60 min with collagenase (2 mg/mL) (Sigma, St. Louis, MO, USA, C1639) at 37 °C; then, the dissociated suspension was first sieved through 100 mesh. The sieved liquid was centrifuged, then suspended and precipitated by PBS, then passed through 200 mesh and 400 mesh sieves. Next, the suspension was washed with RPMI 1640 medium (Gibco, Grand Island, NY, USA, 21870076) and then re-suspended in growth medium with 15% fetal calf serum (Gibco, USA, 10082-147), chick embryo extract (GEMINI, New York, NY, USA, 100-163p), basic fibroblast growth factor (Life, Grand Island, NY, USA, 13256-029) (0.25 µg/100 mL), and RPMI 1640 medium. The suspension was plated on a normal dish and then transferred to a dish coated with matrigel (BD, Franklin Lakes, NJ, USA, 356234) after 2.5 h. The satellite cells were cultured at 37 °C in a cell incubator with 5% CO2 until they converged to 60%. Then, the second differential attachment experiment was performed. The differentiation medium was composed of Dulbecco’s modified eagle medium (DMEM) and 3% (*v*/*v*) horse serum (Gibco, USA). The inhibitor SB273005 (Selleck, Houston, TX, USA, S7540, 10 μM) or DMSO (Sigma) was used to stimulate the satellite cells continuously for 24 h.

**Cell Transfection.** For RNAi assay, the isolated satellite cells were transfected with si-ITGβ6 or si-NC by using Lipofectamine 2000 (Invitrogen, Grand Island, NY, USA, 11668019) in accordance with the manufacturer’s recommendations after the cells converged to approximately 60%. The siRNA and the scrambled negative control were provided by RIBOBIO Biotechnology Co., LTD (Guangzhou, China).

**RNA-seq.** Qualified total RNA was purified by using the RNAClean XP Kit (Beckman Coulter, Inc. Kraemer Boulevard Brea, CA, USA, A63987) and the RNase-Free DNase Set (QIAGEN, Hilden, Germany, 79254). RNA and the library preparation integrity were verified using an Agilent Bioanalyzer 2100 (Agilent technologies, Santa Clara, CA, USA). We accomplished the clustering and first dimension sequencing primer hybridization on cBot of an Illumina sequencing machine in accordance with the cBot User Guide. Sequencing was performed by Shanghai Biotechnology Corporation (Shanghai, China). Edger, which is an R package, was used to screen the DEGs.

**DIA-quantitative proteomics techniques.** This study was completed at Gene Denovo Biotechnology Co., LTD (Guangzhou, China). Cells transfected with si-ITGβ6 and si-NC were selected, with about 10^6^ cells in each group. Triplicate samples were analyzed for each treatment. First, protein extraction was performed with a lysis buffer (containing 1% SDS, 8 M urea, and 1 mg/mL protease inhibitor), followed by protein quality detection and enzymatic hydrolysis, and then pre-treatment by mass spectrometry. By combination with the traditional data dependence acquisition mode, the reference spectrum library was constructed. Then, the mass range of the mass spectrum was divided into several windows using LC-MS/MS technology, and all the ions in each window were fragmented in turn to collect all the sub-ion information, namely, DIA data collection. Quality control of the sequencing data was carried out after the sequencing. The obtained data were then used for protein identification, quantitative, differential protein analysis, and enrichment analysis using Spectronaut analysis software.

**Immunofluorescence.** The satellite cells were transfected with si-ITGβ6, si-PXN, or si-NC when they converged to approximately 50%. Then, these cells were collected when they converged to approximately 80% or induced differentiation for 12 or 36 h (about 10^5^ cells). Data were collected as previously described in Weiya Zhang’s article [19]. The specific steps were as follows: Cells were washed twice with phosphate-buffered saline (PBS), then fixed in 4% paraformaldehyde for 15 min. Then, the satellite cells were washed twice with PBS, incubated in ice-cold 0.25% Triton X-100 at room temperature for 10 min, and again washed thrice. The cells were incubated in blocking solution (3% bovine serum albumin, 0.3% TritonX-100, 10% fetal bovine serum in PBS) at room temperature for 2 h and then incubated in primary antibody at 4 °C overnight. The primary antibodies for immunofluorescence staining are shown in Supplementary Table S1. The cells were washed thrice with PBS and then incubated with anti-mouse IgG (H + L), F (ab’) 2 Fragment (Alexa Fluor^®^ 555 Conjugate) (CST, Boston, MA, USA, 4409), and anti-rabbit IgG (H + L), F(ab’)2 Fragment (Alexa Fluor^®^ 488 Conjugate) (CST, USA, 4412) for 2 h. The cell nuclei were washed thrice with PBS and stained with 4′, 6-diamidino-2-phenylindole (DAPI). Images were captured using an Axio Observer—Inverted microscope system (ZEISS, Oberkochen, Gemany), and 10 random fields were captured for each treatment group.

**Western blot.** The Mammalian Protein Extraction Reagent (Pierce, WA, USA) was used to obtain the protein lysate for approximately 10^6^ cells. A double-plate vertical electrophoresis apparatus (Beijing Liuyi Biotechnology Co., Ltd., Beijing, China, DYCZ-24DH) was used to separate the proteins and transfer protein onto polyvinylidene fluoride membranes (Millipore, St. Louis, MO, USA). Primary antibodies (Supplementary Table S1) and horseradish peroxidase (HRP)-labeled secondary antibodies (Beyotime, Jiangsu, China) were used for immunoblotting. A Tanon 5200 Multi imaging system (Tanon, Shanghai, China) was used to detect the signal produced by the ECL Chemiluminescent HRP Substrate (Millipore, WBKLS0500).

**qRT-PCR.** Total RNA was extracted from about 10^5^ cells. Reverse transcription was performed to initiate cDNA synthesis by using the Prime ScriptTM RT Reagent Kit with gDNA Eraser (TAKARA BIO INC., Otsu, Shiga, Japan). THUNDERBIRD SYBR qPCR Mix (TOYOBO, Tokyo, Japan) was used for qRT-PCR, and the results were monitored using an ABI QuantStudio 6 Real-time fluorescence quantitative PCR system (ABI, Grand Island, NY, USA). Each treatment group contained three biological replicates. β-Tubulin gene was used as the internal control. All primer sequences are listed in the supplementary data (Supplementary Table S2). All results are expressed as the mean ± s.e.m. Unpaired Student’s t-tests were used to determine the statistical significance, and *p* < 0.05 or *p* < 0.01 indicated a significant difference.

## 3. Results

### 3.1. Itgβ6 Is One of the Key Node Genes during Skeletal Muscle Development

**Figure 1:**
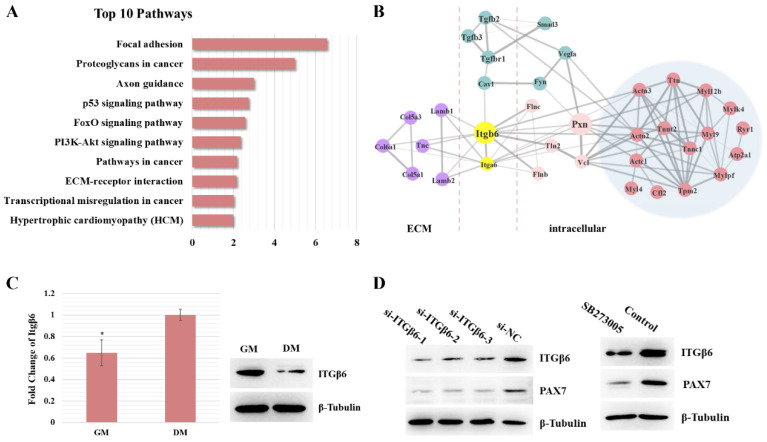
ITGβ6 is involved in the regulation of satellite cell differentiation. (**A**) RNA-seq data were used to analyze the signaling pathways associated with skeletal muscle development. (**B**) A schematic diagram of ITGβ6 interacting with related proteins. (**C**) qRT-PCR and Western blot were performed to detect the expression pattern of the Itgβ6 gene between the proliferation and differentiation status of satellite cells. (**D**) Western blot results of ITGβ6 and PAX7 in proliferative satellite cells when ITGβ6 was inhibited using siRNA or inhibitor. β-Tubulin was used as the internal control for qRT-PCR and Western blot assay, and the relative fold change of qRT-PCR was compared to the expression in proliferative cells. Triplicate samples were analyzed for each treatment, and the results are presented as the mean ± s.e.m. *, *p* < 0.05.

In order to explore the molecular mechanism of satellite cell proliferation and differentiation during skeletal muscle development, we previously studied the development of satellite cells in skeletal muscle at different stages [19]. In the previous study, we isolated satellite cells from mice of different ages for transcriptome sequencing (Week 2, Week 4, Week 6, Week 8, Week 10, and Week 12). Furthermore, differentially expressed genes were clustered into different modules based on WGCNA analysis, among which the genes in the red module were mostly related to differentiation, while the genes in the turquoise module were more related to cell proliferation. Therefore, on the basis of our previous studies, we integrated and analyzed all genes related to cell differentiation and proliferation. Pathways were analyzed using the Kyoto Encyclopedia of Genes and Genomes database (KEGG), and the top ten pathways (FDR < 0.05) are shown in Figure 1A, including focal adhesion, ECM–receptor interaction, the PI3K–AKT signaling pathway, etc. (Figure 1A). Furthermore, the protein interaction relationship was analyzed using STRING software (confidence > 0.7). After filtering, we obtained a simplified interactive network diagram. The network indicated that Itgβ6 acts as a key node connecting ECM-related proteins with cytoskeletal proteins such as filamin (Flnc), vinculin (Vcl), and paxillin (Pxn), and it acts on proteins related to muscle fiber development via the PXN protein (Figure 1B).

Furthermore, the expression level of the Itgβ6 gene during satellite cell differentiation was detected. The real-time quantitative PCR (qRT-PCR) results showed that the mRNA expression levels of Itgβ6 were significantly up-regulated during differentiation, while the Western blotting results showed that the expression levels of the Itgβ6 gene were significantly down-regulated (about 1.7-fold), suggesting that the expression of the Itgβ6 gene is regulated by post-translational modifications during satellite cell differentiation (Figure 1C). In addition, the expression level of PAX7 protein was also detected. RNAi and inhibitor (SB273005) were used to silence the expression of ITGβ6 protein, and the Western blotting results showed that inhibition of ITGβ6 expression resulted in down-regulation of the PAX7 protein level (Figure 1D). These results reveal that Itgβ6 acts as a regulatory factor in skeletal muscle development.

### 3.2. ITGβ6 Is Necessary for the Differentiation of Satellite Cells

**Figure 2:**
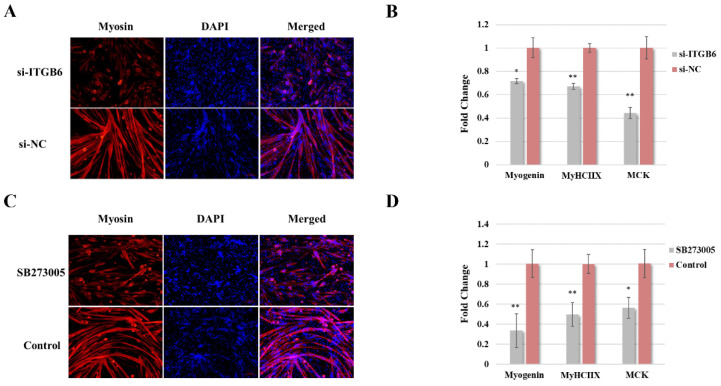
ITGβ6 regulates the myogenic differentiation of satellite cells. (**A**,**C**) Immunofluorescence staining of Myosin (red) in the 36 h differentiated satellite cells when ITGβ6 was inhibited using RNAi or inhibitor, Scan bar: 100 μm. (**B**,**D**) qRT-PCR results of the expression change of Myog, MyHⅡX, and Mck when ITGβ6 was inhibited using siRNA or inhibitor. β-Tubulin was used as the internal control for qRT-PCR, and the relative fold change was compared to the expression in the control group. Triplicate samples were analyzed for each treatment, and the results are presented as the mean ± s.e.m. *, *p* < 0.05; **, *p* < 0.01.

In order to study the function of ITGβ6 in satellite cell differentiation, RNAi technology was performed to inhibit the expression level of ITGβ6 protein in satellite cells, and the role of ITGβ6 in the differentiation of satellite cells was studied via immunofluorescence staining and qRT-PCR. The immunofluorescence staining results revealed that the expression of myosin and the fusion index of myotube were reduced in the si-ITGβ6-transfected group after differentiation for 36 h (Figure 2A). Moreover, the qRT-PCR result revealed that the expression levels of the Myog (Myogenin), MyHCⅡX (Myosin heavy chain ⅡX), and Mck (muscle creatine kinase) genes were decreased in the si-ITGβ6-transfected group (Figure 2B). The inhibition of ITGβ6 through the inhibitor SB273005 exerted similar effects with the small interfering RNA (siRNA) (Figure 2C,D). We verified experimentally that SB273005 had no significant effect on the expression levels of other integrin subtypes at the concentration of 10 uM in satellite cells (data not shown). These results indicate that ITGβ6 is necessary for the maintenance of satellite cell characteristics and the differentiation of satellite cells.

### 3.3. ITGβ6 Is Required to Maintain the Membrane Integrity and Cytoskeleton Stability of Satellite Cells

To further analyze the molecular mechanism by which ITGβ6 regulates satellite cell differentiation, RNAi technology was applied to inhibit the expression level of ITGβ6 protein, followed by a DIA-quantitative proteomics technique to screen differentially expressed proteins (DEPs) regulated by ITGβ6. According to the DIA-quantitative proteomics analysis, a total of 102,198 precursors, 86,995 peptides, 13,709 proteins, and 7698 protein groups were identified (Figure S1A), including 291 DEPs. Compared with the control group, 75 proteins were up-regulated and 216 proteins were down-regulated in the si-ITGβ6 treatment group (Figure S1B,C).

**Figure 3:**
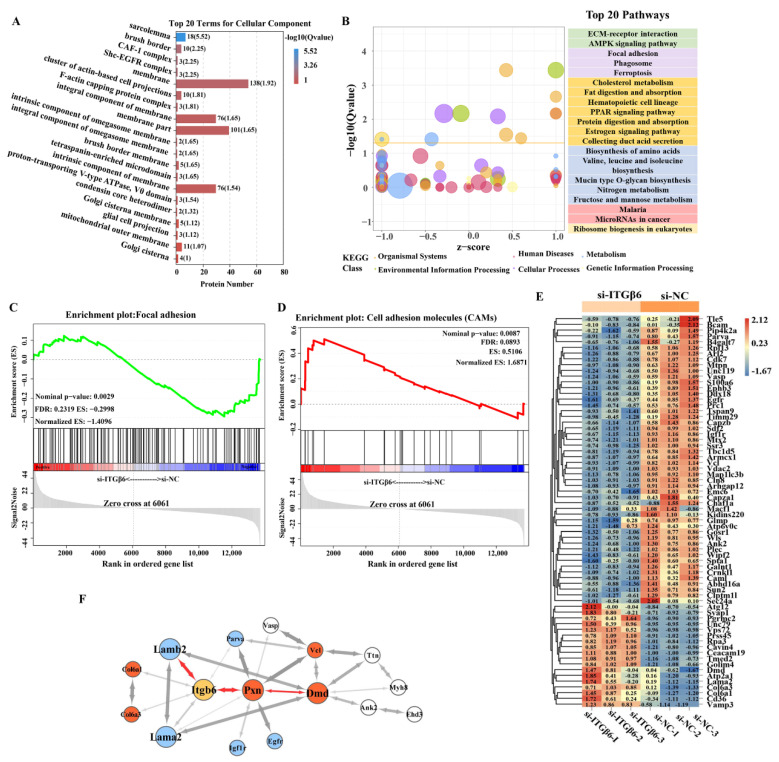
Functional cluster analysis of DEPs. (**A**) The top 20 cellular component terms in GO analysis. (**B**) The top 20 pathways into which differential proteins were enriched. (**C**) GSEA analysis of the focal adhesion pathway. (**D**) GSEA analysis of the cell adhesion molecules (CAMs) pathway. (**E**) Heat map of genes in the top 20 cellular component terms. (**F**) A schematic diagram of protein interactions after filtering.

In addition, GO analysis was performed using the DAVID program (Figure S1D). The biological process analysis showed that DEPs were mainly involved in cellular process, single-organism process, metabolic process, etc. In addition, the molecular function analysis revealed that the DEPs were mainly involved in binding, catalytic activity, structural molecular activity, transporter activity, etc. Remarkably, the enrichment analysis of cell components showed that DEPs were mostly enriched in the membrane part, ECM components, cytoskeleton, and cell junction, including the sarcolemma, cluster of actin-based cell projections, integral component of membrane, tetraspanin-enriched microdomain, etc. (Figure 3A and Figure S1D). Moreover, the pathway analysis results showed that proteins regulated by ITGβ6 were mainly enriched in ECM–receptor interaction, AMPK signaling pathway, focal adhesion, phagosome, etc. (Figure 3B). Furthermore, GSEA analysis demonstrated that ITGβ6 silencing resulted in the suppression of the focal adhesion and adherens junction pathways, as well as the activation of the cell adhesion molecules (CAM) and autophagy—other eukaryotes pathways (Figure 3C,D and Figure S2A,B). These results indicate that the ITGβ6 protein is mainly involved in maintaining cytoskeletal structure and microenvironment stability through regulating membrane proteins, cytoskeleton proteins, and ECM-related proteins.

In order to further explore the key proteins regulated by ITGβ6 in satellite cell development, we focused on the top 20 terms for cellular components. The heat map showed that inhibition of ITGβ6 expression resulted in the down-regulation of membrane-integrity-related proteins and cytoskeleton proteins, while membrane-permeability-related proteins and ECM-related proteins, such as Atg12 and Col6a, tended to be up-regulated (Figure 3E). STRING software was used to analyze the interaction network of proteins in the top 20 pathways. The network showed that ITGβ6 interacts directly with collagen proteins (COL6α1 and COL6α3) and laminin proteins (LAMα2 and LAMβ2) in ECM, and also regulates DMD, VCL, and muscle-fiber-development-related proteins through interaction with PXN (Figure 3F). These results indicate that ITGβ6 is one of the key factors for maintaining the physiological function of satellite cells, and it is necessary for the integrity of the cell membrane and the stability of the cytoskeletal structure.

### 3.4. ITGβ6 Regulates the Differentiation of Satellite Cells through Affecting the Expression of Cytoskeleton Proteins

**Figure 4:**
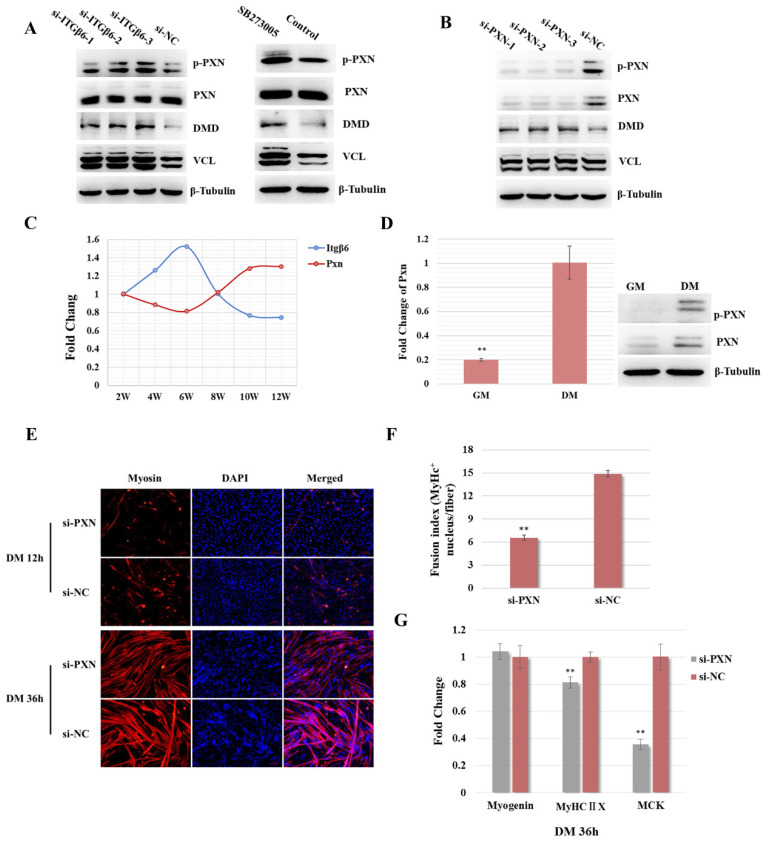
The ITGβ6–PXN pathway regulates the differentiation of satellite cells. (**A**) Western blot was performed to detect the expression levels of PXN, p-PXN, DMD, and VCL when ITGβ6 was inhibited using siRNA or inhibitor. (**B**) Western blot was performed to detect the expression levels of PXN, p-PXN, DMD, and VCL when PXN was inhibited using siRNA. (**C**) qRT-PCR was performed to detect the expression trends of the Itgβ6 and Pxn genes in satellite cells in mice at different ages. (**D**) qRT-PCR and Western blot were performed to detect the expression pattern of the Pxn gene between the proliferation and differentiation status of satellite cells. (**E**) Immunofluorescence staining of Myosin (red) in 12 h or 36 h differentiated satellite cells when PXN was inhibited using RNAi. Nuclei were stained with DAPI (blue). Scale bars: 100 μm. (**F**) Statistical result of myotube fusion ability. Ten random fields were selected to calculate the average number of MyHc+ nuclei in each muscle fiber. **, *p* < 0.01. (**G**) qRT-PCR results of the expression change of Myog, MyHCⅡX, and Mck in the 36 h differentiated satellite cells when PXN was inhibited using siRNA. β-Tubulin was used as the internal control for qRT-PCR and Western blot assay, and the relative fold change of qRT-PCR was compared to the expression in proliferative cells or the control group. Triplicate samples were analyzed for each treatment, and the results are presented as the mean ± s.e.m. **, *p* < 0.01.

In order to further explore the molecular mechanism of ITGβ6 regulating satellite cell differentiation, the key proteins regulated by ITGβ6 were screened. The Western blotting results demonstrated that the inhibition of ITGβ6 by siRNA or inhibitor led to the up-regulation of DMD, VCL, and p-PXN (phosphorylated PXN), but did not affect the expression level of total PXN protein (Figure 4A and Figure S3A). However, inhibition of PXN expression can also up-regulate the DMD and VCL protein expression levels (Figure 4B and Figure S3B). These results suggest that inhibition of ITGB6 could activate PXN, while silencing of both resulted in up-regulation of DMD protein expression.

Furthermore, the effect of PXN on satellite cell differentiation was investigated. Firstly, the expression trend of Itgβ6 and the Pxn gene in satellite cells during skeletal muscle maturation was evaluated. The qRT-PCR results showed that the mRNA level of the Pxn gene was firstly down-regulated slightly and then up-regulated gradually with the development of skeletal muscle, while the expression trend of the Itgβ6 gene was the opposite (Figure 4C). In addition, the expression level of the Pxn gene during the differentiation of satellite cells was detected. The Western blot and qRT-PCR results revealed that the mRNA and protein levels of the Pxn gene were up-regulated during cell differentiation (Figure 4D, about 5-fold). Moreover, immunofluorescence staining results revealed that the expression level of Myosin in the si-PXN transfected group was obviously decreased when satellite cells were induced to differentiate for 12 h. However, when satellite cells were induced to differentiate for 36 h, the number of myotubes in the experimental group was not significantly reduced, while the number of MyHC-positive nuclei in each myotube was significantly reduced, which showed that the fusion ability was significantly inhibited (fusion index > 2-fold) (Figure 4E,F). The qRT-PCR results showed that the expression levels of Myog did not change significantly, but the expression levels of MyHCⅡX and Mck were significantly reduced (Figure 4G). These results indicate that ITGβ6 and PXN are synergistically involved in the regulation of satellite cell differentiation.

### 3.5. ITGβ6 Regulates the Expression of β-Laminin, a Component Protein of ECM

**Figure 5:**
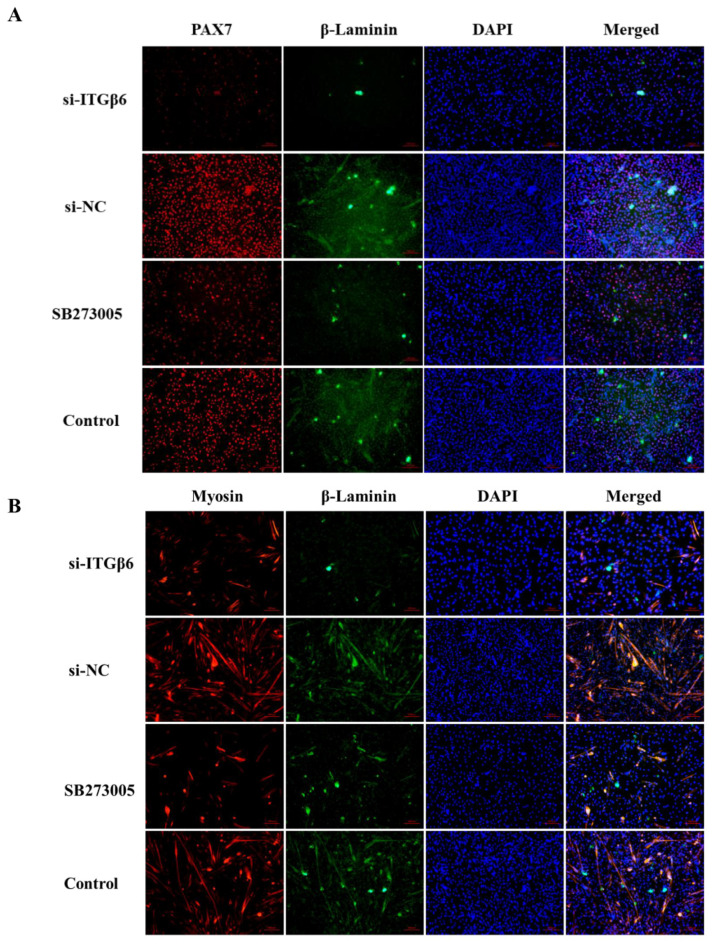
ITGβ6 regulates the expression of β-Laminin. (**A**) Immunofluorescence staining of PAX7 (red) and β-Laminin (green) when ITGβ6 was inhibited using siRNA or inhibitor in proliferative satellite cells. Nuclei were stained with DAPI (blue). Scale bars: 100 μm. (**B**) Immunofluorescence staining of Myosin (red) and β-Laminin (green) when ITGβ6 was inhibited using siRNA or inhibitor in the 12 h differentiated satellite cells. Nuclei were stained with DAPI (blue). Scale bars: 100 μm.

Furthermore, ECM-related proteins interacting with ITGβ6 were screened and verified according to the protein interaction network diagram. The immunofluorescence results showed that the fluorescence signal of β-Laminin protein was significantly weakened after ITGβ6 inhibition during satellite cell proliferation and differentiation (Figure 5A,B). Notably, the immunofluorescence results also showed that the signal of PAX7 protein was distinctly weakened in the inhibition group of ITGβ6, which was consistent with the Western blot result mentioned above (Figure 1D). Interestingly, the fluorescence signal of β-Laminin protein was distinctly enhanced with satellite cell differentiation, indicating that β-Laminin is involved in regulating satellite cell differentiation (Figure 5B).

## 4. Discussion

The normal development and regeneration of skeletal muscle depend on the maintenance of satellite cell properties, such as the abilities of proliferation, differentiation, and self-renewal. As skeletal muscle progenitor cells, satellite cells can not only differentiate into muscle fibers, but also regulate the composition of ECM, thus affecting the development of skeletal muscle [20]. Satellite cells adhere to the basal lamina through the interaction between integrin proteins and ECM-related proteins [14]. Studies have revealed that integrin protein deficiency could lead to intracellular signal transmission disorder and abnormal deposition of ECM proteins, resulting in muscle degeneration and muscular dystrophy [21,22,23].

Accordingly, to reveal the molecular mechanism by which integrin protein regulates skeletal muscle development, we further re-mined the transcriptome data of satellite cells in mice at different stages from our previous study. Moreover, we analyzed the interaction between the genes encoding ECM-related proteins, satellite cell membrane proteins, and cell proliferation- and differentiation-related proteins from the perspective of transcription. Our study demonstrated that Itgβ6 not only acted on ECM proteins (e.g., Col6α1, Lamβ1, Lamβ2, etc.) but also regulated the expression of effector genes in skeletal muscle development through interaction with Pxn. A prior study showed that ITGβ6 protein is involved in regulating the muscle fiber regeneration process [24]. In addition, Qiao et al. demonstrated that the Itgβ6 gene could inhibit the proliferation of porcine satellite cells during skeletal muscle development [25]. Therefore, we further investigated the effect of Itgβ6 on satellite cell differentiation. In line with our expectations, we found that inhibition of ITGβ6 protein expression could effectively inhibit satellite cell differentiation through functional verification, indicating that the ITGβ6 protein was necessary in the process of satellite cell differentiation. Interestingly, expression pattern studies found that the mRNA level of the Itgβ6 gene was up-regulated during satellite cell differentiation, while the protein level was significantly down-regulated. We hypothesized that the expression of the Itgβ6 gene is regulated by post-translational modifications, and ITGβ6 protein might play a regulatory role in differentiation initiation, rather than differentiation maintenance. Furthermore, proteomic analysis showed that inhibition of ITGβ6 expression resulted in suppression of both the TGFβ and MAPK signaling pathways. Studies have shown that the TGFβ [26] and MAPK [27] signaling pathways play important roles in skeletal muscle development, further confirming that ITGβ6 could be involved in regulating satellite cell differentiation.

Remarkably, inhibition of ITGβ6 expression resulted in significant down-regulation of membrane-integrity-related proteins and significant activation of autophagy-related pathways, indicating increased membrane permeability. Therefore, we concluded that ITGβ6 is necessary to maintain the stability of the cell membrane. Interestingly, while the pathways related to cell adhesion were inhibited, the cellular adhesion molecular pathway was activated to some extent. We hypothesize that cells spontaneously initiate other compensation pathways to reduce the damage to cellular physiological functions when membrane function is impaired due to defective expression of ITGβ6. Based on these results, we conclude that ITGβ6 regulates the differentiation ability of satellite cells by influencing the membrane structure and cytoskeletal system.

Moreover, the expression of the PAX7 and β-Laminin proteins was decreased due to the inhibition of the ITGβ6 protein. PAX7 is an important marker of skeletal muscle satellite cells and is necessary for maintaining the characteristics of satellite cells and the expression levels of myogenic regulatory factors [28,29,30]. In previous studies, PAX7 deficiency resulted in a significant decrease in the number of satellite cells and inhibited the expression levels of MYF5, MYOD, and Desmin, which eventually inhibited skeletal muscle regeneration [31,32,33]. In addition, β-Laminin is an important component of the basal membrane and plays an important role in the adhesion of muscle fibers and satellite cells [34]. Deficient expression of Laminin family proteins could result in shedding and atrophy of muscle fibers and inhibition of satellite cell activation and proliferation [14,35,36]. Therefore, we speculated that ITGβ6 could influence the myogenic differentiation potential of satellite cells through regulating the expression level of PAX7 protein, thus affecting terminal differentiation rather than directly participating in the regulation of the effector molecules in the process of myogenic differentiation.

**Figure 6:**
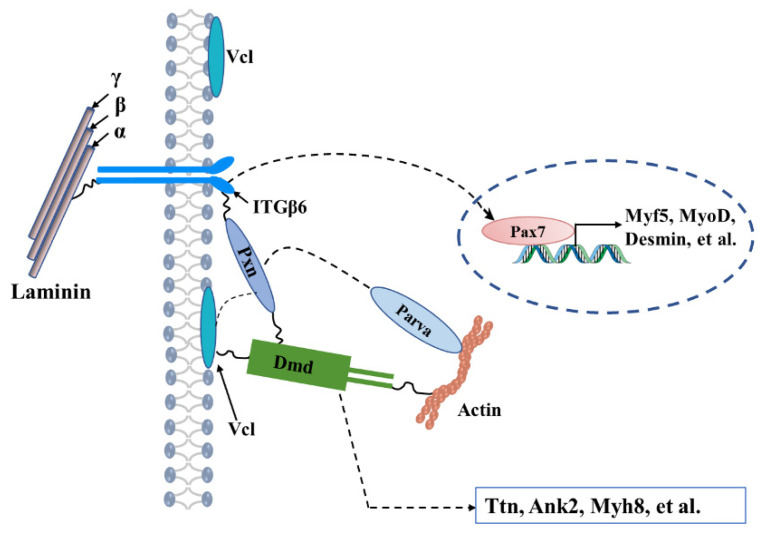
Schematic diagram of ITGβ6 as a key node connecting ECM-related proteins to the cytoskeleton.

According to STRING analysis, we found that PXN might act as an intermediate node between ITGβ6 and DMD, and VCL protein. PXN protein is a component of the cytoskeleton and is involved in cell adhesion [37]. DMD, a part of the dystrophin-glycoprotein complex, bridges actin and the ECM and is required for the development and organization of myofibers as contractile units in striated muscles [38,39,40]. Vinculin is also a cytoskeletal protein associated with the cell–ECM junction [41,42]. Thus, we propose that PXN interacts with the ITGβ6, DMD, and VCL proteins to form a stable scaffold structure to maintain the stability of cell morphology and anchor cells to the basal lamina, thus facilitating the normal physiological functions of satellite cells (Figure 6). Unexpectedly, we found that the expression levels of DMD, VCL, and p-PXN were up-regulated to varying degrees with the down-regulation of the ITGβ6 protein, while the expression levels of DMD and VCL were also upregulated with PXN silencing. We speculate that the cytoskeletal system is a complex network in which defects in one component are compensated by other components to reduce the disturbance to structural stability. However, due to the powerful inhibitory effect of exogenous factors on ITGβ6 expression, this compensation effect was not enough to compensate for the attenuated physiological function of satellite cells.

## 5. Conclusions

In conclusion, ITGβ6 is necessary for skeletal muscle development and is involved in the regulation of satellite cell differentiation and ECM remodeling. The effect of ITGβ6 on satellite cell differentiation mainly occurs before the initiation of differentiation. ITGβ6 regulates the formation of myotubes through influencing the myogenic differentiation potential, membrane integrity, and cytoskeletal system stability of satellite cells.

## Data Availability

The data sets supporting the results of this article were included within the article and additional files. The RNA-seq data are available from the Short Read Archive (SRA) database of NCBI (bio-project accession PRJNA510174, study SRP173522, RNA-seq accessions SRR8453587-SRR8453598). The proteomics data were deposited to the ProteomeXchange Consortium via the PRIDE partner repository with the dataset identifier PXD028650. Project Name: mouse skeletal muscle nano-HPLC-MS/MS. The proteomics data are currently private and can be accessed with a single reviewer account that has been created. Reviewer account details: Username: reviewer_pxd028650@ebi.ac.uk; Password: zKqbOnRG.

## Supplementary Materials

The following supporting information can be downloaded at: https://www.mdpi.com/article/10.3390/life12070926/s1.
