# Supplementary material for: ITGβ6 Facilitates Skeletal Muscle Development by Maintaining the Properties and Cytoskeleton Stability of Satellite Cells
Source: Life (Basel). 2022 Jun 21;12(7):926. doi: 10.3390/life12070926 (PMC9318838; doi:10.3390/life12070926)
Supplement: Supplementary file 1 [file life-12-00926-s001.zip › supplementary figure legends.pdf]

Supplemental Results' Figure Legends

Figure S1. Differential protein analysis using DIA-Quantitative proteomics analysis. A: The number of precursors, peptides, proteins, and protein groups identified. B: The number of up-regulated and down-regulated differential proteins in si-ITGβ6 treated group compared with the negative control. C: Heat map analysis of differential proteins between si-ITGβ6 treated group and negative control. D: Classification secondary bar chart of GO analysis. E: Circular diagram of GO analysis. The outermost layer represents the GO Term, the second layer represents the number of DEPs and the degree of GO Term enrichment, the third layer represents the number of up-regulated and down-regulated proteins, and the fourth layer represents the enrichment factor.

Figure S2: GSEA analysis of Adherens junction, Autophagy-other eukaryotes, TGFβ signaling pathway, and MAPK signaling pathway.

Figure S3: Mean gray value of western blot bands in Figure 4A and B.

Figure S4: Q-PCR results of *Itgβ6* and *Pxn* genes in satellite cells at different age in mice. β-Tubulin was used as the internal control, and the relative fold change was compared to the satellite cells of Week 2 mice. Triplicate samples were analyzed for each treatment, and the results were presented as the mean±s.e.m. *, $P<0.05$; **, $P<0.01$.
