# Supplementary material for: ITGβ6 Facilitates Skeletal Muscle Development by Maintaining the Properties and Cytoskeleton Stability of Satellite Cells
Source: Life (Basel). 2022 Jun 21;12(7):926. doi: 10.3390/life12070926 (PMC9318838; doi:10.3390/life12070926)
Supplement: Supplementary file 1 [file life-12-00926-s001.zip › Table S1.pdf]

Table S1. Primary Antibodies for immunofluorescence and western blot

Primary Antibody	Antigen	Application	Working concentration	Manufacturer	Product code
Anti-Pax7 mouse	PAX7	IF, WB	1:100 dilution	DSHB, USA	
Anti-myosin (skeletal, fast) mouse	myosin	IF	1:200 dilution	Sigma Life Science, USA	M4276
Laminin beta 2 antibody rabbit	β-Laminin	IF	1:400 dilution	Abcam, USA	Ab210956
Phospho-Paxillin Antibody rabbit	p-PXN	WB	1:2000 dilution	ABclonal, P.R.C	AP1156
ITGB6 Antibody rabbit	ITGβ6	WB	1:5000 dilution	ABclonal, P.R.C	A16904
Paxillin Antibody rabbit	PXN	WB	1:5000 dilution	ABclonal, P.R.C	A19100
Anti-Dystrophin antibody (DMD)	DMD	WB	1:1000 dilution	Abcam, USA	ab275391
VCL Rabbit pAb	VCL	WB	1:1000 dilution	ABclonal, P.R.C	A14193
β-tubulin mouse monoclonal antibody	β-tubulin	WB	1:5000 dilution	Biyuntian Biotech, P.R.C	AF1216
