# Supplementary material for: ITGβ6 Facilitates Skeletal Muscle Development by Maintaining the Properties and Cytoskeleton Stability of Satellite Cells
Source: Life (Basel). 2022 Jun 21;12(7):926. doi: 10.3390/life12070926 (PMC9318838; doi:10.3390/life12070926)
Supplement: Supplementary file 1 [file life-12-00926-s001.zip › Table S2.pdf]

Table S2. Primers for Q-PCR in this study

Name	Sequence (5' - 3')
MyHCIIX-F	GGACCCACGGTCGAAGTTG
MyHCIIX -R	CCCGAAAACGGCCATCT
Mck-F	GCTTATGGTGGAGATGGAGA
Mck-R	GGCCATCACGGACTTTTATT
Myogenin-F	CAATGCACTGGAGTTCGGT
Myogenin-R	CTGGGAAGGCAACAGACAT
β-tubulin-F	GACTATGGACTCCGTTCGCTC
β-tubulin-R	TATTCTTCCCGGATCTTGCTG
Itgβ6-F	GGGGGTGTCACTGGCGAT
Itgβ6-R	GAGGATTGGTTCCCGTTTGC
Pxn-F	TCCAGAAGGCTTCCACGAGA
Pxn-R	CTGCCCGTCGTGCTCAAA
