# Supplementary figures and images for: ITGβ6 Facilitates Skeletal Muscle Development by Maintaining the Properties and Cytoskeleton Stability of Satellite Cells
Source: Life (Basel). 2022 Jun 21;12(7):926. doi: 10.3390/life12070926 (PMC9318838; doi:10.3390/life12070926)

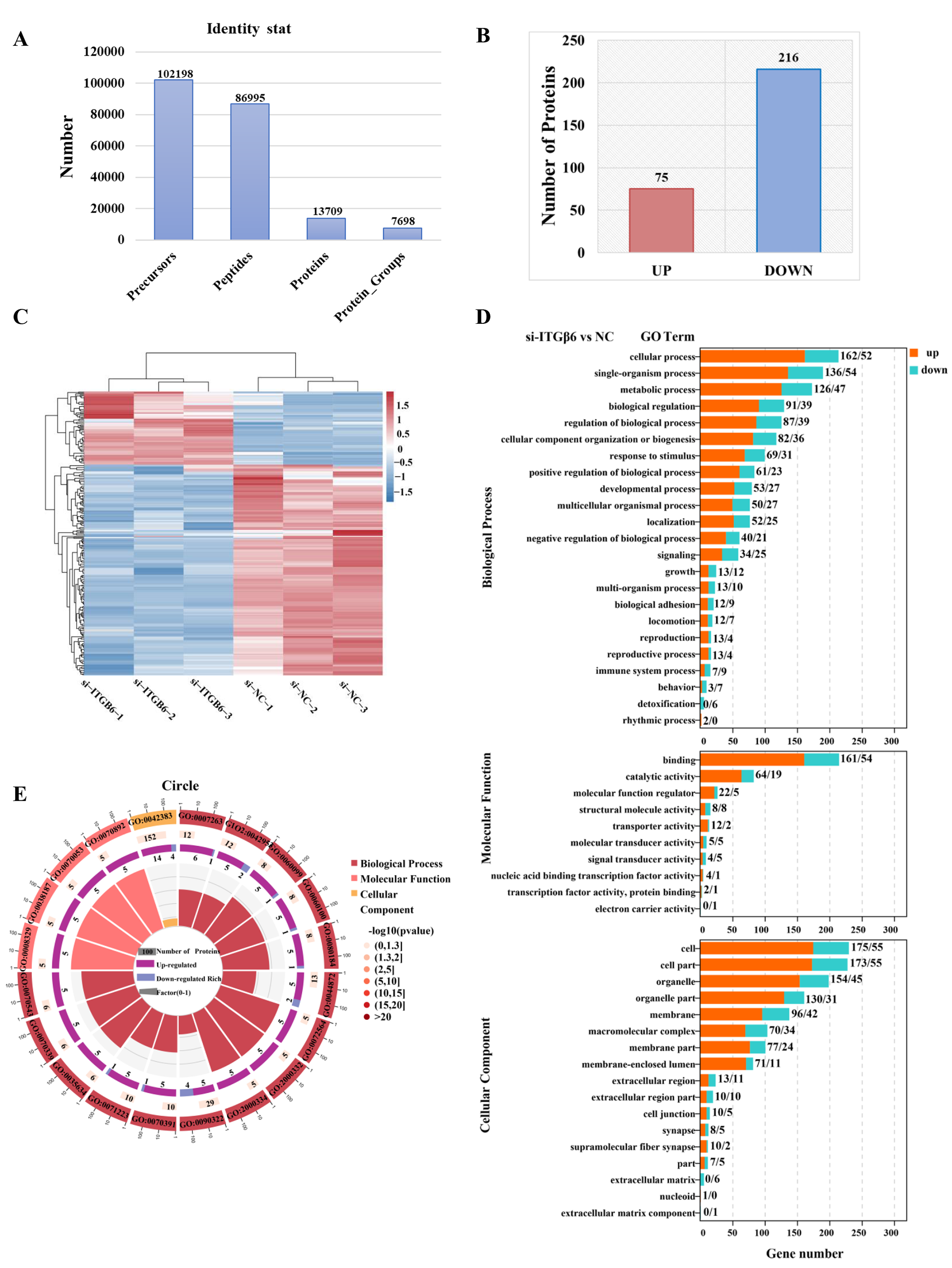

Supplement: Supplementary file 1 [file life-12-00926-s001.zip › Figure S1.tif]

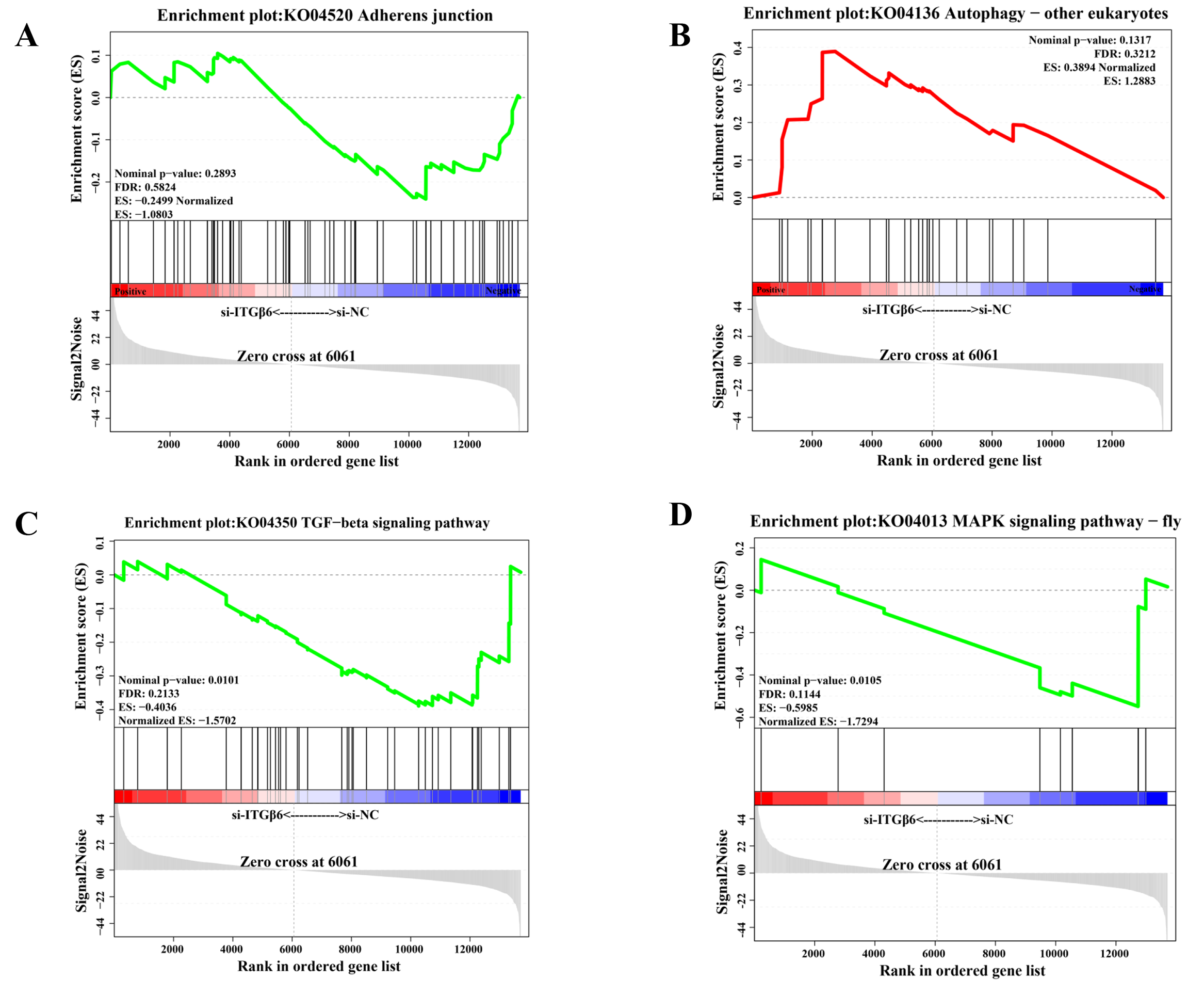

Supplement: Supplementary file 1 [file life-12-00926-s001.zip › Figure S2.tif]

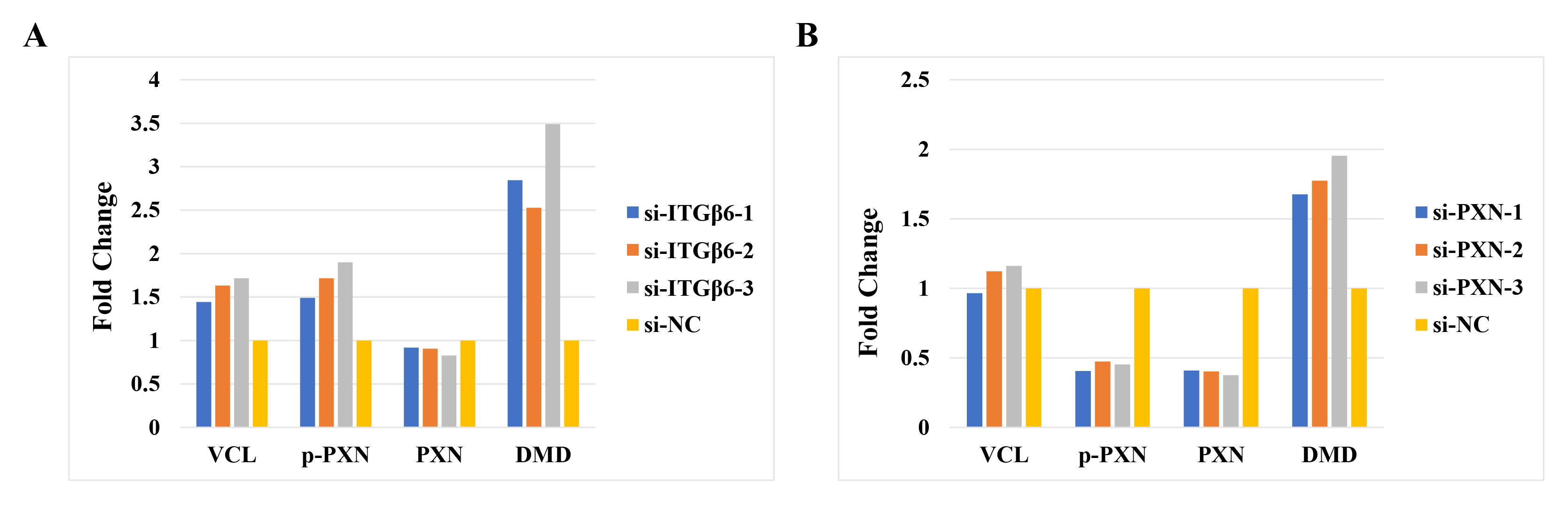

Supplement: Supplementary file 1 [file life-12-00926-s001.zip › Figure S3.tif]

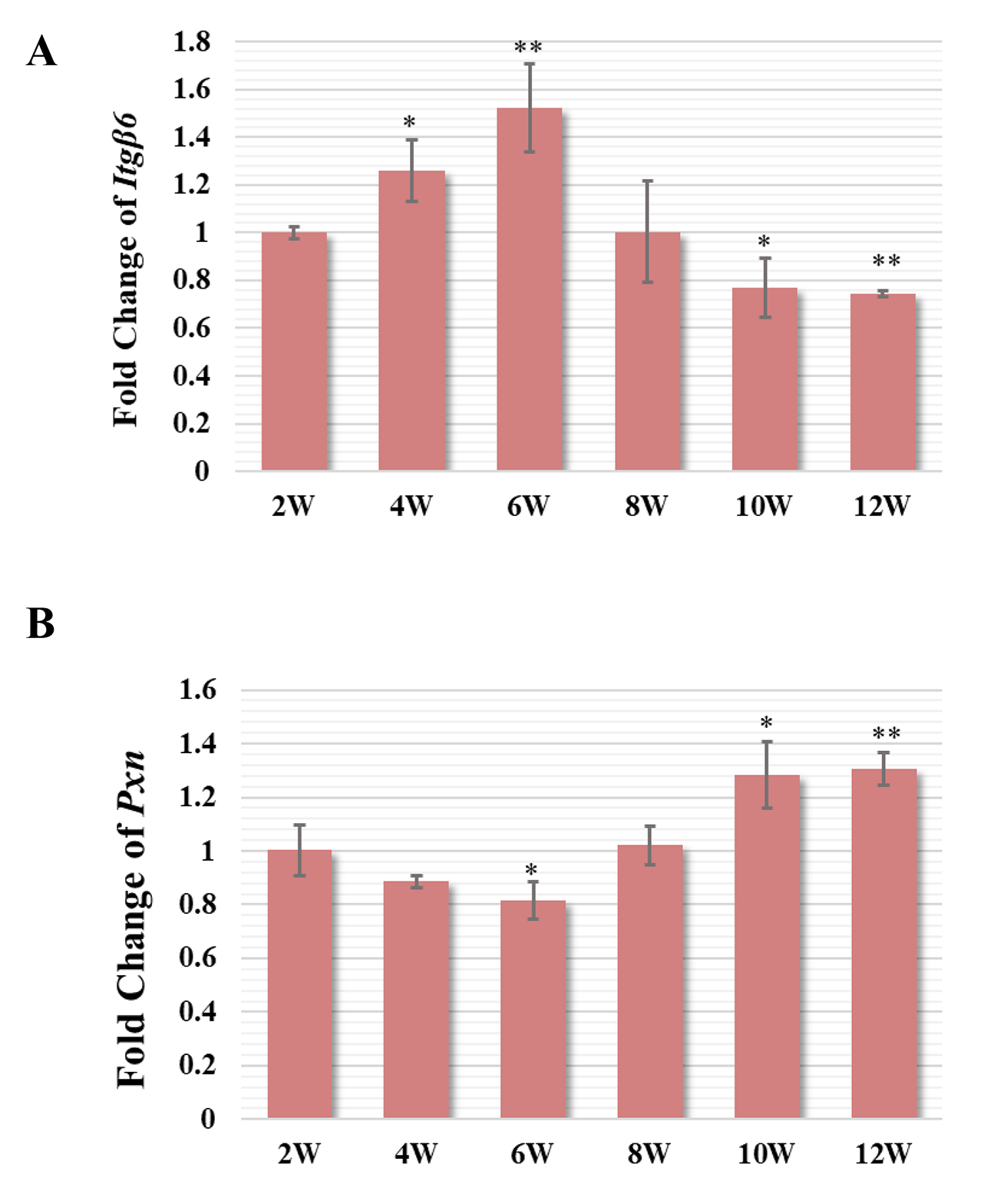

Supplement: Supplementary file 1 [file life-12-00926-s001.zip › Figure S4.tif]
